# Image restoration in frequency space using complex-valued CNNs

**DOI:** 10.3389/frai.2024.1353873

**Published:** 2024-09-23

**Authors:** Zafran Hussain Shah, Marcel Müller, Wolfgang Hübner, Henning Ortkrass, Barbara Hammer, Thomas Huser, Wolfram Schenck

**Affiliations:** ^1^Center for Applied Data Science, Faculty of Engineering and Mathematics, Bielefeld University of Applied Sciences and Arts, Bielefeld, Germany; ^2^Biomolecular Photonics Group, Faculty of Physics, Bielefeld University, Bielefeld, Germany; ^3^CITEC—Center for Cognitive Interaction Technology, Bielefeld University, Bielefeld, Germany

**Keywords:** image restoration, image denoising, super-resolution, convolutional neural networks (CNNs), complex-valued convolutional neural networks (CV-CNNs), complex-valued attention gates, structured illumination microscopy, Fast Fourier Transform

## Abstract

Real-valued convolutional neural networks (RV-CNNs) in the spatial domain have outperformed classical approaches in many image restoration tasks such as image denoising and super-resolution. Fourier analysis of the results produced by these spatial domain models reveals the limitations of these models in properly processing the full frequency spectrum. This lack of complete spectral information can result in missing textural and structural elements. To address this limitation, we explore the potential of complex-valued convolutional neural networks (CV-CNNs) for image restoration tasks. CV-CNNs have shown remarkable performance in tasks such as image classification and segmentation. However, CV-CNNs for image restoration problems in the frequency domain have not been fully investigated to address the aforementioned issues. Here, we propose several novel CV-CNN-based models equipped with complex-valued attention gates for image denoising and super-resolution in the frequency domains. We also show that our CV-CNN-based models outperform their real-valued counterparts for denoising super-resolution structured illumination microscopy (SR-SIM) and conventional image datasets. Furthermore, the experimental results show that our proposed CV-CNN-based models preserve the frequency spectrum better than their real-valued counterparts in the denoising task. Based on these findings, we conclude that CV-CNN-based methods provide a plausible and beneficial deep learning approach for image restoration in the frequency domain.

## 1 Introduction

Convolutional neural networks (Krizhevsky et al., 2017) have become the leading state-of-the-art algorithms in the field of computer vision, ranging from image classification, pattern recognition, and object detection to tasks such as image restoration. Several image restoration problems, such as image denoising (Zhang et al., 2018; Li et al., 2020), reconstructing super-resolution (SR) image data (Dong et al., 2014), image inpainting (Xie et al., 2012), and JPEG compression artifact removal (Zheng et al., 2018) have been extensively investigated using CNN architectures. These CNN methods have demonstrated substantial improvements in image restoration tasks over classical approaches (Lim et al., 2017; Gao et al., 2024; Zuo et al., 2018; Tran et al., 2020; Gu et al., 2012; He et al., 2010; Timofte et al., 2013, 2014). The vast majority of CNN based image restoration techniques have been developed by employing standard real-valued convolutional neural networks (RV-CNNs). RV-CNN based architectures are constrained to work with real-valued inputs, outputs, and parameters. In various disciplines such as biomedical engineering, physics, optics, radar and telecommunications, however, signals are recorded that are complex valued in their raw form (Schreier and Scharf, 2010; Barrachina et al., 2021). Complex representations of signals have real and imaginary components to e.g. carry magnitude and phase values that provide additional insights which are otherwise difficult to capture with purely real-valued signals (Foreman, 2012). Also, in the frequency domain, the contents of an image are represented as complex numbers (Xu et al., 2020).

Complex-valued convolutional neural networks (CV-CNNs) have recently been proposed to deal with such complex signals (Benvenuto and Piazza, 1992; Georgiou and Koutsougeras, 1992). CV-CNNs rely on complex inputs, network parameters, and the outputs are either complex or real values depending on the task (Lee et al., 2022). Since the real and imaginary components of a complex signal contain more information, the use of complex-valued networks is more crucial compared to its real-valued counterpart (Bassey et al., 2021). In image classification (Hafiz et al., 2015), segmentation (Akramifard et al., 2012), speech enhancement (Tsuzuki et al., 2013), and MRI fingerprint signal processing (Virtue et al., 2017), CV-CNNs have shown exceptional performance compared to their real counterparts. However, the performance of CV-CNNs in the frequency domain has not been extensively investigated in the context of image restoration problems such as image denoising and super-resolution image reconstruction. Quan et al. (2021) investigated the potential of CV-CNNs for image denoising using dummy imaginary values in the spatial domain. Rawat et al. (2021) denoised chest X-ray (CXR) images by using the complex-valued based method with residual learning. Similarly, Pham et al. (2021) evaluated the performance of a complex Fourier network for the image denoising problem by explicitly convolving a complex filter with the Fourier transform of the corrupted image. Nevertheless, CV-CNNs have never been directly employed for the denoising and reconstructing of super-resolution structured illumination microscopy (SR-SIM) and other conventional images in the frequency domain.

Deep learning algorithms based on real-valued networks have revolutionized the denoising and reconstruction of high-quality SR-SIM images. Structured illumination microscopy (SIM) is an extensively used super-resolution imaging technology due to its ability to double the spatial resolution for live cell imaging beyond the diffraction limit of light (Heintzmann and Huser, 2017; Schermelleh et al., 2019; Demmerle et al., 2017). SR-SIM technology is considered the most straightforward super-resolution (SR) reconstruction method among various other SR approaches since it requires fewer raw SIM samples with low illumination intensity levels (Ströhl and Kaminski, 2016; Zheng et al., 2021). However, the conventional SR-SIM reconstruction algorithms require high SNR raw SIM samples to reconstruct the high-quality SR-SIM images (Huang et al., 2018). Consequently, the reconstruction algorithms are unable to produce high-quality SR-SIM images from raw samples acquired under low SNR conditions (Smith et al., 2021). Several RV-CNN based methods have been developed to denoise and reconstruct the SR-SIM images (Shah et al., 2021; Jin et al., 2020; Chen et al., 2021; Shah et al., 2022). Given that image restoration tasks are challenging and ill-posed problems, this leads to the existence of numerous viable possible solutions in the high-dimensional space during inference (Belthangady and Royer, 2019). Furthermore, the RV-CNNs follow the spectral bias within the Fourier spectrum, learning the low-frequency modes faster than the high-frequency modes (Rahaman et al., 2019). As a result of these concerns, it has been discovered that the SR-SIM images denoised and reconstructed using real-valued deep-learning approaches lose part of the high-frequency information (Shah et al., 2021; Qiao et al., 2021). The gap in the high-frequency information is highly visible in the Fourier spectra of the restored SR-SIM images. As a result, the disparity in the Fourier spectrum of the restored and reference images raises various concerns about the effectiveness of RV-CNNs-based image restoration approaches.

### 1.1 Motivation and contributions

The primary motivation behind this work is to retrieve a restored image that exhibits a Fourier spectrum closely resembling the Fourier spectrum of the reference or ground truth image. There has been very scarce research conducted in this particular context. Qiao et al. (2021) proposed a deep Fourier channel attention network (DFCAN) to overcome the frequency content difference across high-frequency information using Fourier channel attention (FCA) mechanism in the spatial domain. Similarly, Liu et al. (2023) investigated and minimized the gap in the high-frequency components by proposing a dual-domain learning strategy for the reconstruction of SIM images. However, their architecture requires repetitive transformation of features within each block, which impedes the exploration of the full potential of CV-CNNs. This prompted us to initiate additional research in this area, resulting in the formulation of two research questions to investigate this topic:

1. Is it possible to perform image denoising and super-resolution entirely in the frequency domain using CV-CNNs?
2. Do CV-CNNS outperform RV-CNNs in terms of generalization performance when applied to previously unseen SR-SIM modalities?

To address these research problems, we propose CV-CNN based image restoration algorithms for denoising and reconstruction of SR-SIM images in the frequency domain rather than the pure spatial domain. This work investigates the full capabilities of CV-CNNs for image restoration problems, intending to reduce the Fourier spectrum difference between the reconstructed and reference SR-SIM images. This work makes the following significant contributions:

- We explore the potential of CV-CNNs and suggest a novel complex-valued attention gate (ℂ-AG).
- We propose two CV-CNN based architectures, named complex-valued collaborative attention network (CV-CAN) and complex-valued dual domain attention network (CV-DDAN), both equipped with complex-valued attention gates for the denoising task.
- Comparison of the proposed CV-CNN with existing RV-CNN architectures for the denoising and knowledge transfer tasks.
- A pure CV-CNN based architecture named complex-valued super-resolution network is designed for the super-resolution task in the frequency representation.

Moreover, we also demonstrate that our proposed complex-valued based denoising architecture CV-DDAN surpasses real-valued CNN based approaches visually as well as in terms of peak-signal-to-noise ratio (PSNR) and structural similarity index measurement (SSIM) values. Similarly, the Fourier spectrum of the resulting denoised images by CV-CNN based approaches is more promising than their real-valued counterparts. We test our proposed architectures on SR-SIM images as well as the commonly used BSD500 benchmark datasets. The results indicate that our proposed methods are not limited to super-resolution microscopic images, but also perform well-across other datasets.

## 2 Materials and methods

This section covers the key components of complex-valued neural networks and the Fast Fourier Transform algorithm used in this work. In addition, the mechanism of the incorporated attention gates is also discussed in this section. Finally, the publicly available datasets are also described in this section. The Red-fairSIM and UNet-fairSIM architectures have already been described in our prior work (Shah et al., 2021) and will not be reiterated here in detail. The Red-fairSIM and UNet-fairSIM methods are based on the combination of fairSIM (Müller et al., 2016) with the RedNet (Mao et al., 2016) and UNet (Ronneberger et al., 2015) architectures (Shah et al., 2021). Both RedNet and UNet methods are constructed using RV-CNNs (Shah et al., 2021).

### 2.1 Complex-valued neural network components

This subsection provides an overview of the complex components, including the complex-valued convolutional layer, activation function, and max pooling.

#### 2.1.1 Complex-valued convolution layer

The complex number has both real and imaginary components in its representation. The complex-valued convolution (CV-Conv) layer have been designed to deal with complex values. The implementation of CV-Conv layer involves four real-valued convolution layers. In CV-Conv layer, a complex filter matrix *K* = *K*_ℜ_+*iK*_ℑ_ is convolved (*) with a complex input matrix *I* = *I*_ℜ_+*iI*_ℑ_ where the individual variables *K*_ℜ_, *K*_ℑ_, *I*_ℜ_, *I*_ℑ_ are real matrices, considering a vector can be represented as a matrix with one of two dimension being one. The ℜ and ℑ denote the real and imaginary components. The convolution operator is distributive in the complex domain, hence the convolution of the kernel *K* with the input *I* is following:


(1) $$\begin{array}{l} K*I=\left(I_{ℜ}*K_{ℜ}-I_{ℑ}*K_{ℑ}\right)+i\left(I_{ℜ}*K_{ℑ}+I_{ℑ}*K_{ℜ}\right) \end{array}$$

**Figure 1:**
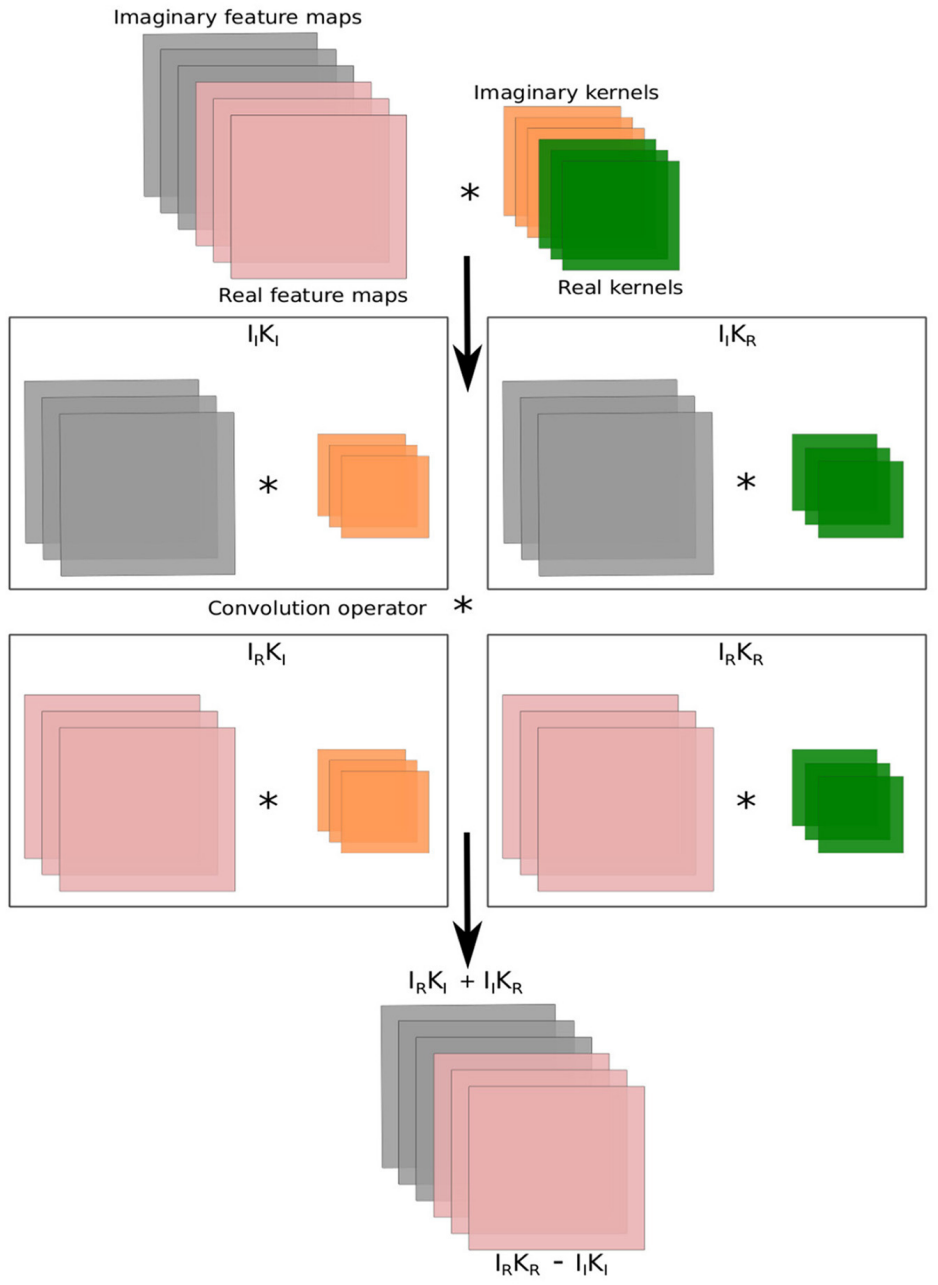
Computations in the complex-valued convolution layer. The symbol ^*^ represents the convolution operation.

Figure 1 illustrates the complex-valued convolution operation. Similarly, according to Ding and Hirose (2013), we can write the matrix notations for the real and imaginary parts of the convolution operation as follows:


(2) $$\begin{array}{l} \left[\begin{array}{c} ℜ\left(K*I\right)\\ ℑ\left(K*I\right) \end{array}\right]=\left[\begin{array}{cc} K_{ℜ} & -K_{ℑ}\\ K_{ℑ} & K_{ℜ} \end{array}\right]*\left[\begin{array}{c} I_{ℜ}\\ I_{ℑ} \end{array}\right] \end{array}$$

The implementation of CV-Conv can be represented by Equation 2. The CV-Conv learns by backpropagation, a sufficient condition is to use a loss function as well as activations that are differentiable with respect to real and imaginary components separately in the network (Hirose and Yoshida, 2012; Chiheb et al., 2017). The authors in Hirose and Yoshida (2012) demonstrate that the separately differentiable functions are compatible with the backpropagation of the CV-Conv layer.

#### 2.1.2 Complex-valued activations

A variety of activation functions have been created to handle complex-valued representations. In this work, we used the complex ReLU (ℂReLU) activation function (Chiheb et al., 2017). The ℂReLU activation function applies the ReLU individually to the real and imaginary parts of the neuron.


(3) $$\begin{array}{l} \mathbb{C}ReLU\left(I\right)=ReLU\left(I_{ℜ}\right)+iReLU\left(I_{ℑ}\right) \end{array}$$

The Equation 3 shows the mathematical form of the ℂReLU activation function, *I*_ℜ_, and *I*_ℑ_ represents the real and imaginary feature components of the input feature *I*. ℂReLU is a split-complex activation function, and the real and imaginary components are both individually and sectionally differentiable (Chiheb et al., 2017).

#### 2.1.3 Complex-valued Max pooling

The complex-valued max pooling is the implementation of conventional max pooling individually on the real and imaginary part of the complex-valued features in the network (Barrachina et al., 2022) as shown in Equation 4.


(4) $$\mathbb{C}\text{Max(I)}={\text{Max(I}}_{ℜ})+i{\text{Max(I}}_{ℑ})$$

The ℂMax-pooling in the complex-valued CNN works with the max-by-magnitude approach in Equation 4. The real and imaginary parts are individually processed in this layer, likewise in the other layers (Chiheb et al., 2017).

### 2.2 Fast Fourier Transform

The Fast Fourier Transform (FFT) is used to obtain frequency information from images by transforming them from the spatial domain to the complex domain. FFT is a fast and efficient algorithm for computing a signal's discrete Fourier transform (DFT) or its inverse (Popa and Cernăzanu-Glăvan, 2018). The symmetry and periodic properties of FFT reduces the computational complexity. For an image of size *N*×*N*, (*p, q*) and (*u, v*) denote the spatial and frequency coordinates. Correspondingly, *I*(*p, q*) and *I*(*u, v*) represent the spatial and frequency values. The 2D DFT is defined as


(5) $$\begin{array}{l} F\left[I\left(u,v\right)\right]=\overset{N-1}{\underset{p=0}{\sum }}\overset{N-1}{\underset{q=0}{\sum }}I\left(p,q\right)e^{-i2\pi \left(\frac{u.p}{N}+\frac{v.q}{N}\right)} \end{array}$$

Since FFT (ℱ) is a bijective function in the image space that means we can define the inverse of FFT (ℱ^−1^) as follows:


(6) $$\begin{array}{l} F^{-1}\left[I\left(p,q\right)\right]=\frac{1}{N^{2}}\overset{N-1}{\underset{u=0}{\sum }}\overset{N-1}{\underset{v=0}{\sum }}I\left(u,v\right)e^{i2\pi \left(\frac{u.p}{N}+\frac{v.q}{N}\right)} \end{array}$$

Equations 5, 6 demonstrate that an image can be transformed back and forth from spatial to frequency domain without losing any information (Gonzalez, 2009).

### 2.3 Attention gates

**Figure 2:**
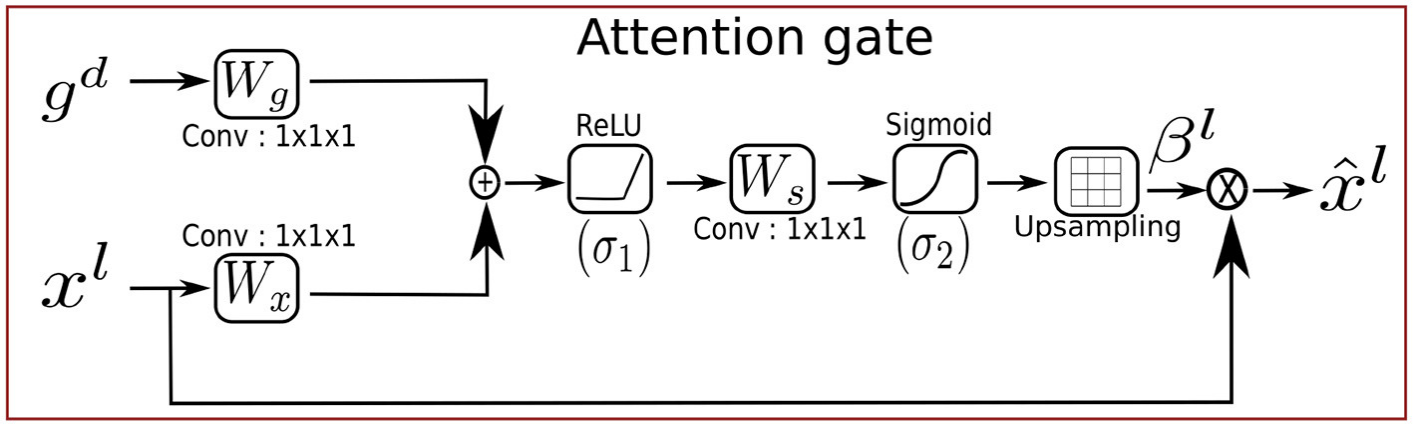
Schematic of the attention gate (AG).

Attention gates (AGs) are widely used attention mechanisms in the field of natural language processing (NLP) (Li et al., 2018) and computer vision for segmentation (Zuo et al., 2021), image captioning (Huang et al., 2019), and classification (Wang et al., 2017). They are categorized into two types: Hard attention (Mnih et al., 2014) involves reinforcement learning and is non-differentiable, which makes the training of the model more complicated. Soft attention (Jetley et al., 2018) is probabilistic and can be trained by using gradient descent and utilizing standard back-propagation without implementing Monte Carlo sampling. Oktay et al. (2018) proposed Attention-UNet by incorporating the attention gates (AGs) into the concatenation based skip connections of the standard UNet (Ronneberger et al., 2015) architecture to extract pancreas segmentation in 3D abdominal CT images. The architecture of Attention-UNet is shown in the upper branch of Figure 4A. The main concept behind AGs is to emphasize the salient features which are propagated through the skip connections. The AGs focus on the relevant information while suppressing the irrelevant information from the background region such as noise (Oktay et al., 2018). The AG takes two input vectors, ${\text{x}}^{l}\in {\mathbb{R}}^{F_{l}\to H_{x}\times W_{x}\times C_{x}}$ and ${\text{g}}^{d}\in {\mathbb{R}}^{F_{d}\to H_{g}\times W_{g}\times C_{g}}$, representing the input feature vector and a gating signal vector where *F*_*l*_ and *F*_*d*_ corresponds to the number of feature maps in layers *l* and *d* with spatial dimensions height (H), width (W), and number of channels (C), respectively, as illustrated in Figure 2. The features of the gating signal selected from the coarser scale of the deeper layers to suppress the irrelevant information. The input feature maps are downsampled to the resolution of gating signal prior to the AGs. The output ${\hat{\text{x}}}^{l}\in {\mathbb{R}}^{H_{x}\times W_{x}\times C_{x}}$ of the AGs is the element-wise multiplication of the attention weight vector $\beta^{l}\in {\mathbb{R}}^{H_{x}\times W_{x}\times C_{x}}$ with the input feature vectors **x**^*l*^ as shown in Equation 7. The attention weights identify the relevant image regions and preserve only the meaningful activation which are related to the task.


(7) $$\begin{array}{l} {\hat{\text{x}}}^{l}={\text{x}}^{l}\cdot \beta^{l} \end{array}$$

The gating vector is combined with the input vector by additive attention (Yu et al., 2018) instead of multiplicative attention.


(8) $$\beta^{l}=\sigma_{2}(W_{s}^{T}(\sigma_{1}(W_{x}^{T}x^{l}+W_{g}^{T}g^{d}+b_{g}))+b_{s}))$$

In Equation 8, the linear transformations are performed by: $W_{x}\in {\mathbb{R}}^{F_{l}\times F_{s}},W_{g}\in {\mathbb{R}}^{F_{d}\times F_{s}}$ and $W_{s}\in {\mathbb{R}}^{F_{s}\times 1}$, and bias terms ${\text{b}}_{g}\in {\mathbb{R}}^{F_{s}}$ and **b**_*s*_∈ℝ. *F*_*s*_ is the number of feature maps in the intermediate convolution layer *s*. The linear transformations are generated by using 1 × 1 × 1 channel-wise convolution layers to decouple the feature maps from higher to lower dimensional space for the gating operation at each image scale, which reduces the training parameters and computational cost of the AGs. The linear transformation is followed by the sigmoid activation function $\sigma_{2}\left({\text{x}}^{l}\right)=\frac{1}{1+\exp\left(-{\text{x}}^{l}\right)}$, and the ReLU activation function $\sigma_{1}\left({\text{x}}^{l}\right)=max\left(0,{\text{x}}^{l}\right)$ on each feature vector. Finally, the attention weights are upsampled to the dimensions of **x**^*l*^ using trilinear interpolation before the element-wise multiplication. These operations are carried out prior to the concatenation function to ensure that only significant and relevant information is included. The gradients of the background regions are down-weighted in the backward pass. The Attention-UNet combines the power of attention gates with UNet to guide the denoising process by selectively focusing on structural features in the image while suppressing the noise and irrelevant details. However, the attention gates are designed with real-valued components, they are limited to real-valued features and cannot be used effectively with the complex-valued features since complex-valued features cannot be fully captured by the real-valued gating mechanism.

### 2.4 Datasets

In this study, we used three different types of datasets: two microscopic datasets, primarily Tubulin and Vesicle datasets, in addition to a non-microscopic BSD dataset. SIM microscopy (Gustafsson, 2000) was used to obtain raw SIM images of Tubulin and Vesicle datasets under different illumination levels and microscopic settings. The raw Tubulin and Vesicle SIM samples were acquired using 3D SIM imaging technologies (Gustafsson et al., 2008). The stack of 15 raw SIM images (i.e., five phases and three orientations) of dimensions 15 × 512 × 512 (frames × width × height) pixels of both SIM structures were propagated into the reconstruction algorithm to produce a super-resolution SIM image of size 1024 × 1024 (width x height) pixels. The fairSIM reconstruction algorithm (Müller et al., 2016) and the softWoRx commercial software were used to reconstruct the Tubulin and Vesicle SR-SIM samples in the frequency domain from the raw SIM images. The Tubulin dataset consists of 101 fields-of-view (FOVs), each FOV recorded for 200 timestamps. The Vesicle dataset is reconstructed using the 3D-SR-SIM method (i.e., softWoRX software), resulting in 3D-SR-SIM samples. However, each z-plane of the 3D-SR-SIM data is extracted using a slice-by-slice approach as 2D sample. The slicing strategy yields 175 FOVs (i.e., basically 175 different *z*-planes extracted from nine FOVs originally), each FOV is acquired for different timestamps. Tubulin and Vesicle datasets contain 2,525 and 7,284 samples, respectively. The training and test sets of both datasets were partitioned based on the number of FOVs. The training sets for the Tubulin and Vesicle datasets were built using 81 and 121 FOVs, respectively. The training sets of the Tubulin and Vesicle datasets compromise 2,025 and 5,562 samples. The remaining samples from both datasets were used for the test sets. Each sample in both datasets consists of the reconstructed 16-bit noisy SR-SIM input and a reference image of size 1, 024 × 1, 024 (width × height) pixels. The input images of both datasets contain real noise, which is a combination of mixed Poisson-Gaussian noise along with the reconstruction artifacts. The complete characteristics, description, and sample preparation of both datasets are further explained in the Shah et al. (2023). The Berkeley segmentation dataset (BSD500), which is related to conventional image restoration problems, was selected as the third dataset in this work (Martin et al., 2001). The input images in this dataset were corrupted by the introduction of additive white Gaussian noise with zero mean and standard deviation of 30, respectively.

## 3 Proposed model architectures

This section will cover all the proposed methodologies used in this work to explore the scope of complex-valued CNN based image restoration networks, as well as the novel complex-valued attention gate that is employed in conjunction with the proposed complex-valued denoising and super-resolution architectures.

### 3.1 Fourier transform encoding and decoding layers

To transform an image from the spatial to the frequency domain, we used a Fourier transform encoding (FTE) layer which is based on the FFT algorithm. The FFT algorithm is used by the FTE layer to convert the input image or feature maps from the spatial domain to the complex representation. The FTE layer also shifts the DC component of FFT feature representation to the center-most location, where the positive and negative frequencies surround the DC component. Since CV-CNNs only interact with the complex values, meaningful complex-valued feature mappings must be transmitted into the CV-CNN layer for further processing.

The Fourier transform decoding (FTD) layer reverses the complex-valued feature representations into their real values using the inverse Fast Fourier transform (IFFT). The DC component of the Fourier representation is first shifted from the center-most location back to the original position, along with the low and high-frequencies. Following that, the IFFT is applied to complex-valued feature maps to convert them back into real-valued spatial feature maps. These real-valued feature maps are then propagated to the RV-CNNs. These FFT-based encoding and decoding layers are used within our proposed architectures.

### 3.2 Complex-valued attention gates

**Figure 3:**
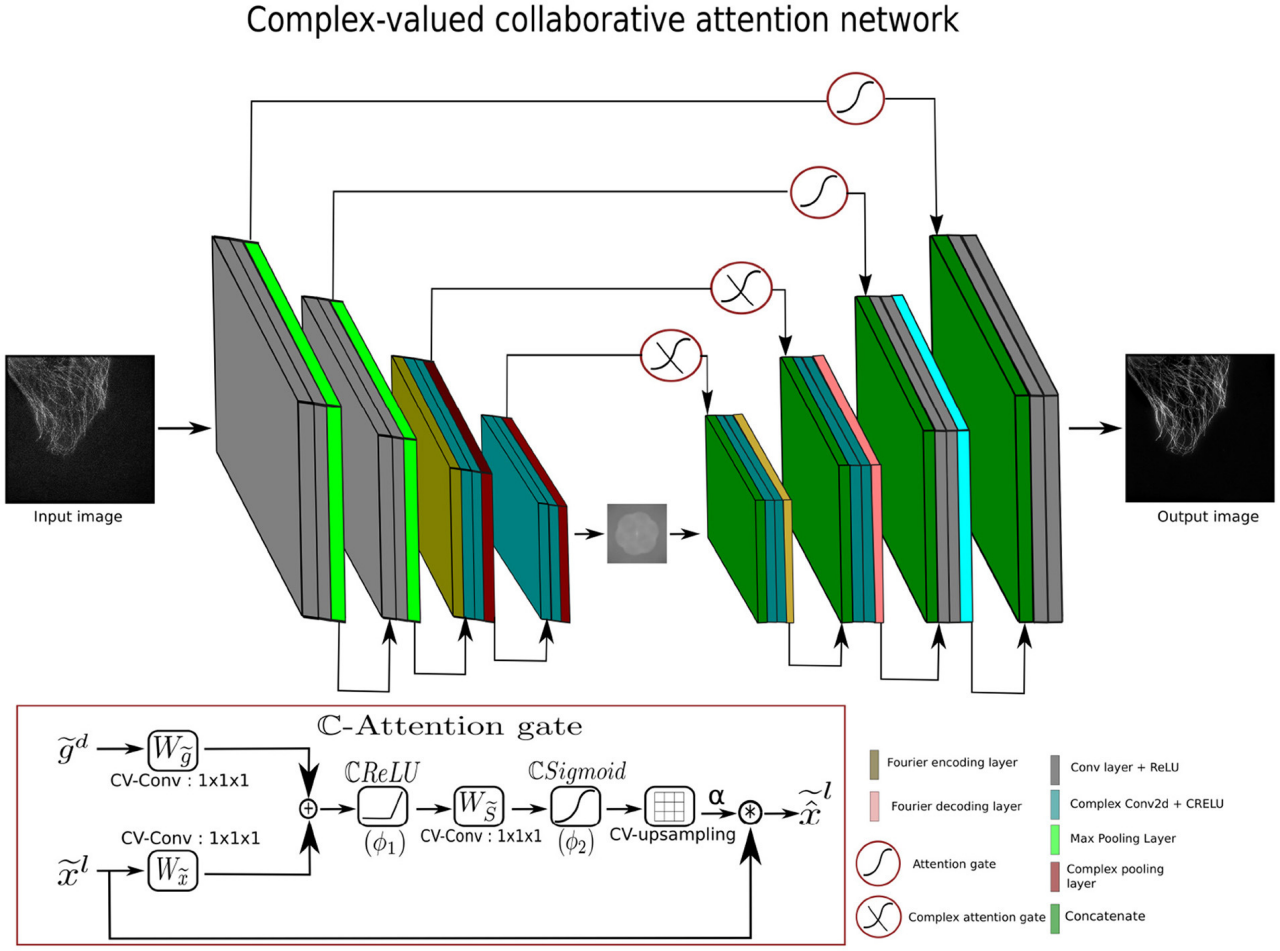
Architecture of complex-valued collaborative attention net.

The standard attention gates (AGs) are not designed to compute attention values natively with complex features. One possibility is to process only the real part (i.e., magnitude information) of feature maps in the attention gates, while discarding the imaginary part (i.e., phase information). However, the loss of phase information can often have significant consequences, since the imaginary part carries a crucial structural representation in the complex-valued features. To mitigate the loss of phase information, complex-valued attention gates (ℂ-AGs) have been developed during this work. The ℂ-AGs inspired by the real-valued AGs are one of the major contributions of this work; their core architecture resembles real-valued attention gates. To handle the complex-valued feature maps, the ℂ-AG is built around complex components. The complex components in the ℂ-AGs allow the attention mechanism to capture both real and complex values of the input features and to leverage the phase information in the attention mechanism of the skip connections of complex-valued networks. The complex attention weight vector in ℂ-AGs, enables the attention mechanism to recognize the salient frequency regions in the frequency domain while suppressing the irrelevant frequency regions. The inputs to ℂ-AGs are the complex input feature vector and the complex gating signal vector, ${\tilde{\text{x}}}^{l}\in {\mathbb{C}}^{F_{l}\to H_{\tilde{x}}\times W_{\tilde{x}}\times C_{\tilde{x}}}$ and ${\tilde{\text{g}}}^{d}\in {\mathbb{C}}^{F_{d}\to H_{\tilde{g}}\times W_{\tilde{g}}\times C_{\tilde{g}}}$, where *F*_*l*_ and *F*_*d*_ represent the feature maps with dimensions height (H), width (W), and number of channels (C) of layers *l* and *d*, respectively, as shown in Figure 3. The tilde indicates the complex-valued representation. The complex gating signal is selected from a coarser scale. Since ℂ-AGs only work with the complex-valued feature maps, the convolution layer in the complex attention block is replaced by CV-Conv layers. The input and the gating vectors are propagated through the CV-Conv layers and summed element-wise to maximize the aligned weights while minimizing the unaligned weights (Oktay et al., 2018). The resulting vector goes through a ℂReLU (ϕ_1_), 1x1x1 CV-Conv layer, and ℂSigmoid (ϕ_2_) activation function to produce the complex attention weights (α). The complex attention weights are upsampled to the dimension of ${\tilde{\text{x}}}^{l}$ using a complex-valued upsampling layer, as shown in Figure 3.


(9) $$\begin{array}{l} \alpha^{l}=\phi_{2}\left(W_{\tilde{s}}^{T}\left(\phi_{1}\left(W_{\tilde{x}}^{T}{\tilde{\text{x}}}^{l}+W_{\tilde{g}}{\tilde{\text{g}}}^{d}+{\text{b}}_{\tilde{g}}\right)\right)+{\text{b}}_{\tilde{s}}\right) \end{array}$$

In Equation 9, $W_{\tilde{s}}\in {\mathbb{C}}^{F_{s}\times 1},W_{\tilde{x}}\in {\mathbb{C}}^{F_{l}\times F_{s}},W_{\tilde{g}}\in {\mathbb{C}}^{F_{d}\times F_{s}}$ are the linear transformations with bias terms ${\text{b}}_{\tilde{g}}\in {\mathbb{C}}^{F_{s}}$ and ${\text{b}}_{\tilde{s}}\in \mathbb{C}$. *F*_*s*_ represents the number of feature maps in the intermediate layer $\tilde{s}$.

In the final step, the output of the ℂ-AGs is obtained by the convolution (*) of the complex-valued feature maps with the complex-valued attention weight vectors rather than the element-wise multiplication, due to their complex nature, as shown in the Equation 10.


(10) $$\begin{array}{l} {\tilde{\hat{\text{x}}}}^{l}={\tilde{\text{x}}}^{l}*{\text{α}}^{l} \end{array}$$

In Equation 10, ${\tilde{\hat{\text{x}}}}^{l}\in {\mathbb{C}}^{H_{\tilde{x}}\times W_{\tilde{x}}\times C_{\tilde{x}}}$ is the complex attention gate output of complex feature maps ($\tilde{\text{x}}$) and complex attention weights (α^*l*^). The proposed ℂ-AGs are trainable and differentiable, and thus fall in the category of soft attention.

### 3.3 Complex-valued attention UNet

**Figure 4:**
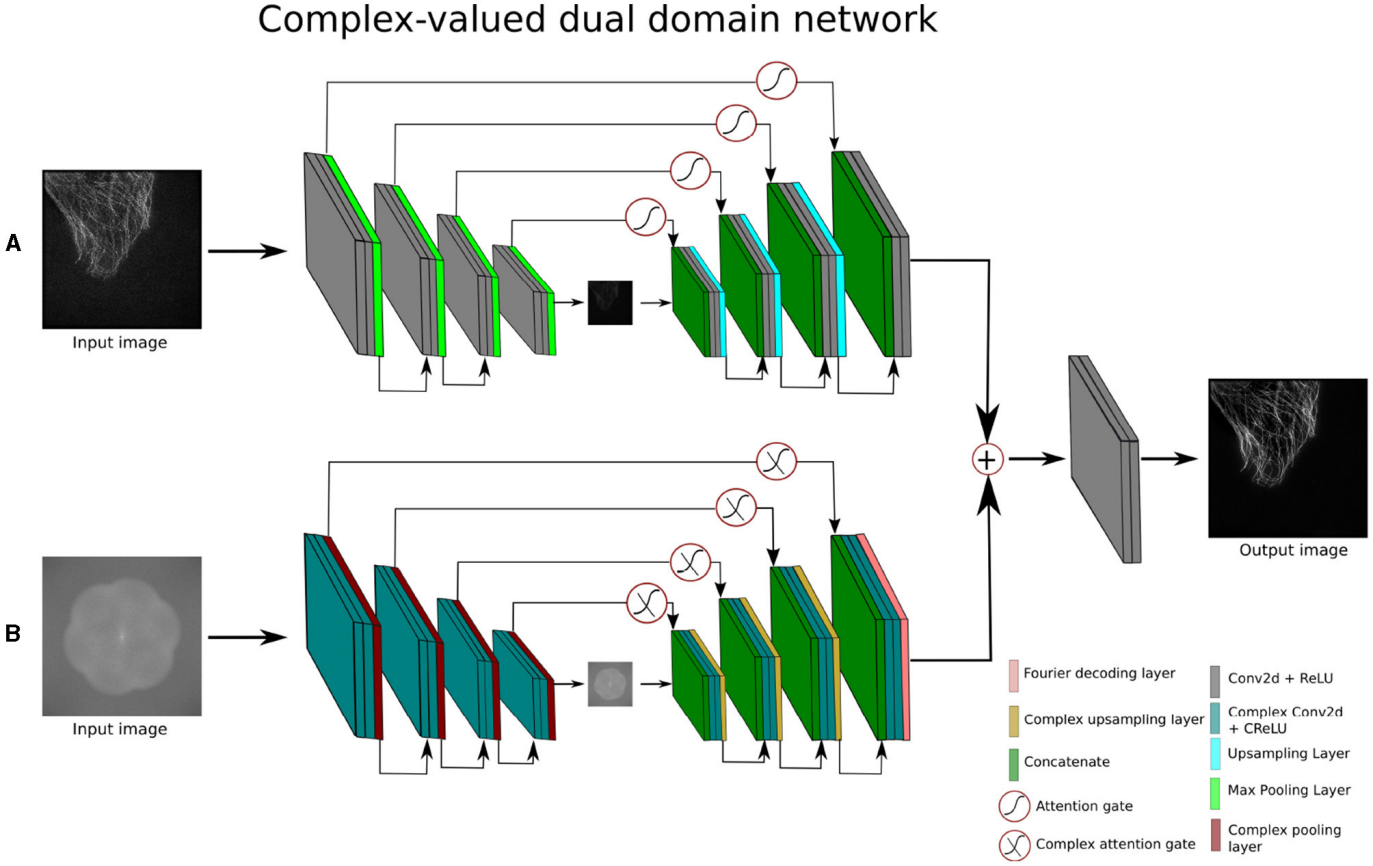
Architecture of complex-valued dual domain attention network **(A)** is the spatial branch and **(B)** depicts the complex branch of the network.

To denoise and reconstruct the super-resolved images entirely in the complex/frequency domain, we devise the underlying Attention-UNet (Oktay et al., 2018) to a complex-valued Attention-UNet (CV-Atten-UNet). The CV-Atten-UNet is a complex version of the real-valued Attention-UNet since the model is constructed with complex components such as CV-convolution, ℂmax pooling, and ℂattention gates (ℂ-AGs) to interact with the complex features. Consequently, the input images are transformed into complex inputs ($I_{in}\in {\mathbb{C}}^{m_{1}\times m_{1}}$) using a Fourier encoding layer. The complex components incorporate the complex-valued representations (i.e., the real and imaginary information) to enhance the effectiveness of the complex-valued networks (Lee et al., 2022). Similarly, the deployment of ℂ-AGs allows the network to dynamically adjust the feature fusion, leading to the capture of adaptive feature representations by suppressing the irrelevant features. ℂ-AGs connect the skip connection symmetrically to the adjacent complex encoder and decoder blocks, as shown in the lower branch of Figure 4B. The architecture is based on four complex encoder and decoder blocks. Each complex encoder block contains two complex-valued convolution layers along with ℂRelu activation functions and a complex pooling layer to compress the complex feature maps at different scales. The complex decoder block expands the features and is made up of two complex-valued convolution layers along with a complex-valued upsampling layer. In the final block, the Fourier decoding layer is merged with a single convolution layer to obtain the spatial output ($O\in {\mathbb{R}}^{m_{1}\times m_{1}}$). The description of CV-Atten-UNet is given in Equation 11.


(11) $$\begin{array}{l} \text{Output}=\text{FTD}\left({\text{Complex decoder}}_{n}\left({\text{Complex encoder}}_{n}\left(I_{in}\right)\right)\right) \end{array}$$

where:

Complex encoder block : CV-Conv → ℂReLU → CV-Conv → ℂReLU → ℂMax pooling
Complex decoder block : CV-Conv → ℂ*ReLU* → CV-Conv → ℂRelu → ℂUpsampling
FTD : Represents the Fourier transform decoding layer.

In the Equation 11, *n* is 4, reflecting the number of complex encoder and decoder blocks in the contraction and expansion sections.

### 3.4 Complex-valued collaborative attention network

To enhance the performance of CV-CNN-based methods, we propose a complex-valued collaborative attention network (CV-CAN) to eliminate noise and provide a high-quality clean image. Figure 3 shows the architecture of CV-CAN which takes the spatial input image ($I_{in}\in {\mathbb{R}}^{m_{1}\times m_{1}}$) and reconstructs a spatial output ($O\in {\mathbb{R}}^{m_{1}\times m_{1}}$). Specifically, the CV-CAN network is comprised of several real and complex-valued encoder-decoder blocks. The real-valued encoder blocks first compress the image and extract the low level features. The features maps produced by the real-valued encoder blocks are converted from spatial into frequency domain. The transformation of the feature maps takes place using the FTE layer. The complex-valued encoder blocks extract further features in the frequency domain. The encoder blocks exploit different level of details in the spatial and frequency domains. Similarly, the complex-valued decoder blocks combines the low and high level to reconstruct the desired complex-valued feature maps. The final output image is obtained by employing the real-valued decoder blocks followed by the FTD layer. The real and complex encoder-decoder blocks are composed of real and complex-valued components, respectively. The proposed ℂ-AGs are also employed in the skip connections across the adjacent complex-valued blocks to emphasize the relevant significant features in the frequency domain. Similarly, the real-valued encoder-decoder blocks are also joined via the skip connections with attention gates to focus on the significant and relevant spatial features. The learnable AGs compress the irrelevant noisy background information (Oktay et al., 2018).

The CV-CAN employs two complex and real-valued encoder decoder blocks. The description of real and complex blocks is illustrated in Equation 12.


(12) $$\begin{array}{l} \text{Output}={\text{Decoder}}_{n}(\text{FTD}({\text{Complex decoder}}_{n}({\text{Complex encoder}}_{n}\\ \;(\text{FTE}({\text{Encoder}}_{n}(I_{in})))))) \end{array}$$

where:

Encoder block : Conv → ReLU → Conv → ReLU → Max pooling
Decoder block : Conv → ReLU → Conv → Relu → Upsampling

Complex encoder and decoder blocks are explained in Equation 11. The FTE and FTD correspond to the Fourier transform encoder and decoder layers. The n represents the number of blocks, which is 2.

### 3.5 Complex-valued dual-domain attention network

The complex-valued dual-domain attention network (CV-DDAN) operates on both spatial and frequency domain data simultaneously to further improve the frequency learning ability of the existing CV-CNN methods. The network accepts two inputs ($I_{in1}\in {\mathbb{R}}^{m_{1}\times m_{1}}$ and $I_{in2}\in {\mathbb{C}}^{m_{1}\times m_{1}}$), from two different representations, one of which is a real-valued spatial input and the other is a complex-valued frequency input as shown in the Figure 4. The input of the complex branch is generated by the FTE layer. To process these inputs, the network has two separate encoding-decoding pathways to extract the features from spatial and complex domains. The adjacent real and complex encoder-decoder blocks of the dual-domain attention network are joined with each other via real and complex-valued AGs in their respective skip connections. The output of the complex branch is transformed from the complex feature to the real feature maps using the FTD layer, as stated in Equation 13. The outputs of both branches are fused by an additive operation and fed into the final output block to produce a spatial domain output image ($O\in {\mathbb{R}}^{m_{1}\times m_{1}}$).

The purpose of the dual-domain attention network is to capture and exploit maximum information from the spatial and frequency domains by using real and complex branches during the training process. Another important aspect is to retrieve the high frequency components of the output image which is not the case with traditional CNN methods.


(13) $$\begin{array}{l} \text{Output}={\text{Decoder}}_{n}({\text{Encoder}}_{n}(I_{in1}))+\text{FTD}({\text{Complex decoder}}_{n}\\ \;({\text{Complex encoder}}_{n}(I_{in2}))) \end{array}$$

The description of real and complex encoder and decoder blocks is illustrated in the Equations 11 and 12, where the value of *n* is 4.

### 3.6 Complex-valued super-resolution network

**Figure 5:**
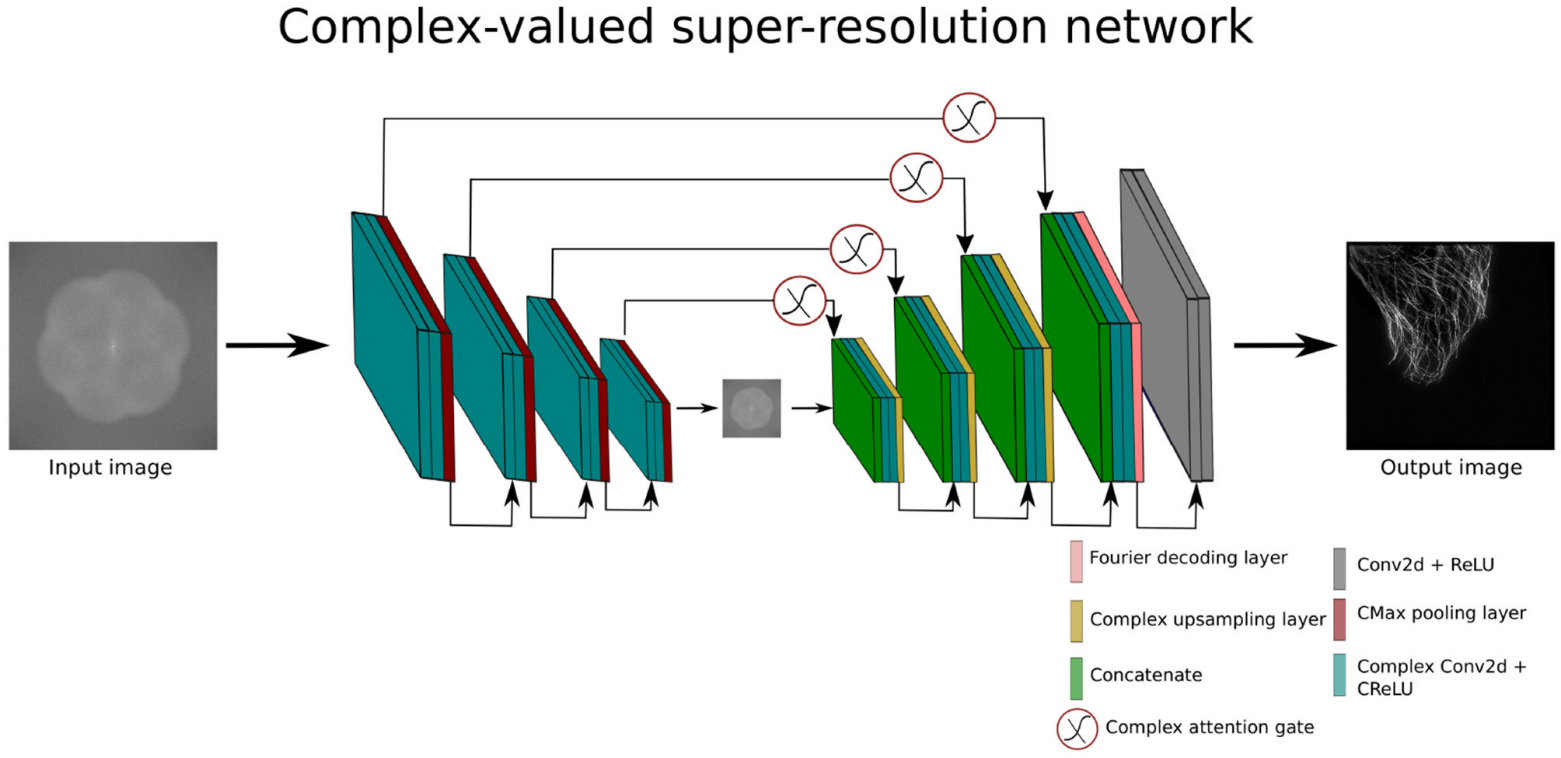
Architecture of complex-valued super-resolution network.

To investigate the potential of CV-CNNs for the super-resolution task in the frequency domain we design a complex-valued super-resolution network (CV-SRN). The CV-SRN model is the modified version of CV-Att-UNet model. The integration of an upsampling block into the CV-Att-UNet architecture results in the CV-SRN model. The main concept behind this architecture is to explore the reconstruction capabilities of CV-CNN models in the complex domain after the application of Fourier Transform. CV-SRN converts the complex-valued feature maps before the upsampling block using the FTD layer as shown in Figure 5.

The CV-SRN architecture takes complex-valued input ($I_{in}\in {\mathbb{C}}^{m_{1}/2\times m_{1}/2}$) which is half the size of the final output image ($O\in {\mathbb{R}}^{m_{1}\times m_{1}}$). The CV-SRN processes the low resolution image of size 512 × 512 pixels and generates a high-quality SR-SIM of size 1, 024 × 1, 024 pixels. This architecture can effectively increase the spatial dimensions of super-resolution images by a factor of two.

## 4 Experimental results

We carried out a series of experiments to assess the performance of the proposed CV-CNN architectures in denoising and super-resolution tasks. Of special importance is the comparison with the corresponding real-valued counterparts. Furthermore, this study also investigates the generalization capabilities of the suggested denoising methods.

We used three publicly available datasets for the benchmarking, two microscopic ones (Tubulin, Vesicle) and one conventional (BSD). The Tubulin and Vesicle datasets consist of high-quality SR-SIM images with real noise, which is a mixture of Poisson-Gaussian noise (Shah et al., 2023), while the BSD dataset contains additive white Gaussian noise (AWGN). To ensure fair comparison, all the denoising networks were trained on these three datasets with consistent hyper-parameters such as number of epochs, loss function, and learning rate^1^. The mean squared error loss function and the ADAM optimizer were used to train all the networks.

### 4.1 Denoising

**Figure 6:**
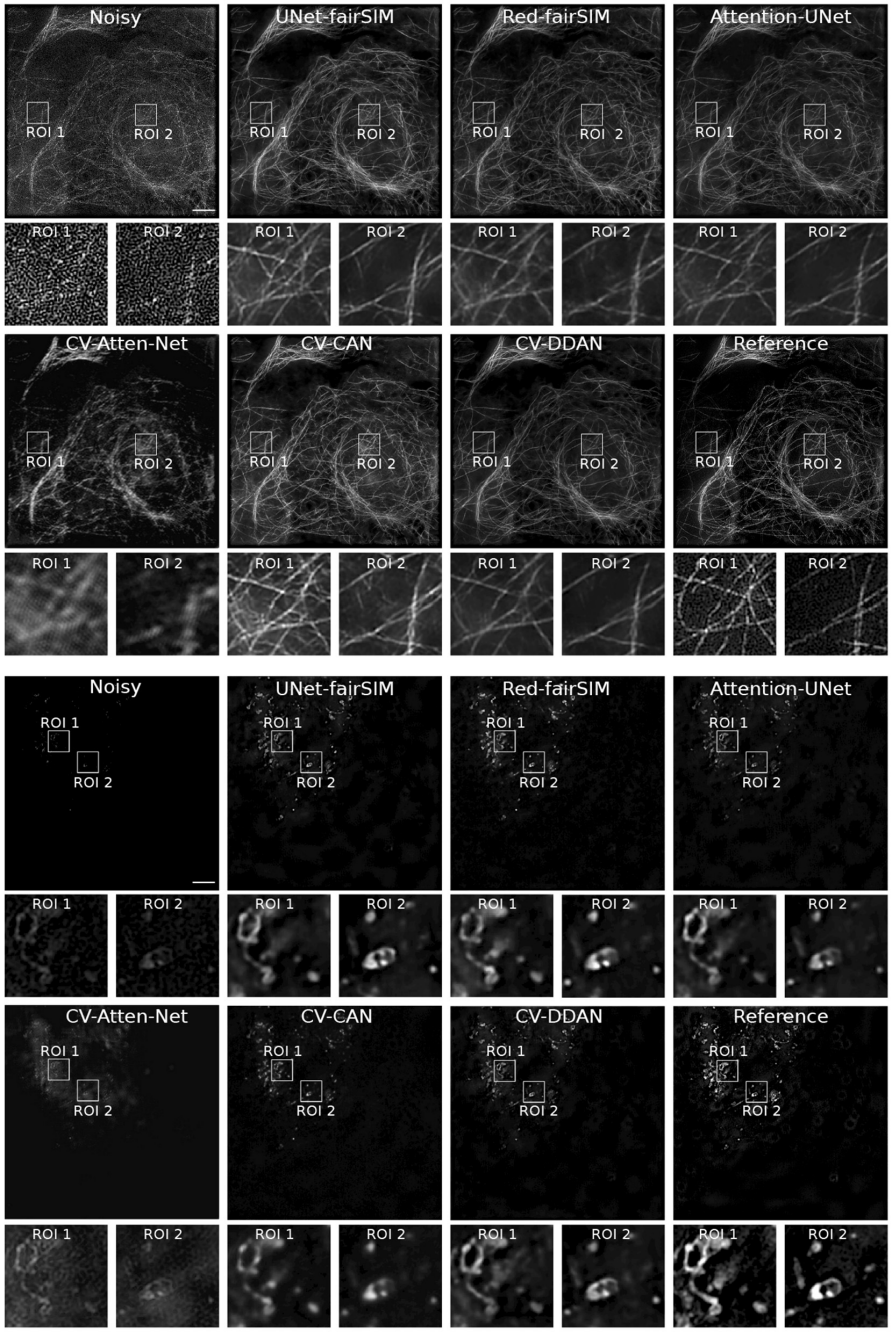
Results of test samples of Tubulin and Vesicle datasets. The first four rows show the results of the Tubulin images and the next four rows depict the Vesicle SR-SIM samples. The first and fifth rows contain the noisy as well as the denoised images of RV-CNN based methods, whereas, the third and seventh rows display the images denoised by CV-CNN based models along with the reference images. The second, fourth, sixth, and eight rows display two regions of interest (ROI) extracted from each respective image. The cropped ROIs of size 100 × 100 pixels are upsampled to 300 × 300 for demonstration purposes. SR-SIM images scale bar: 4μ*m*.

**Figure 7:**
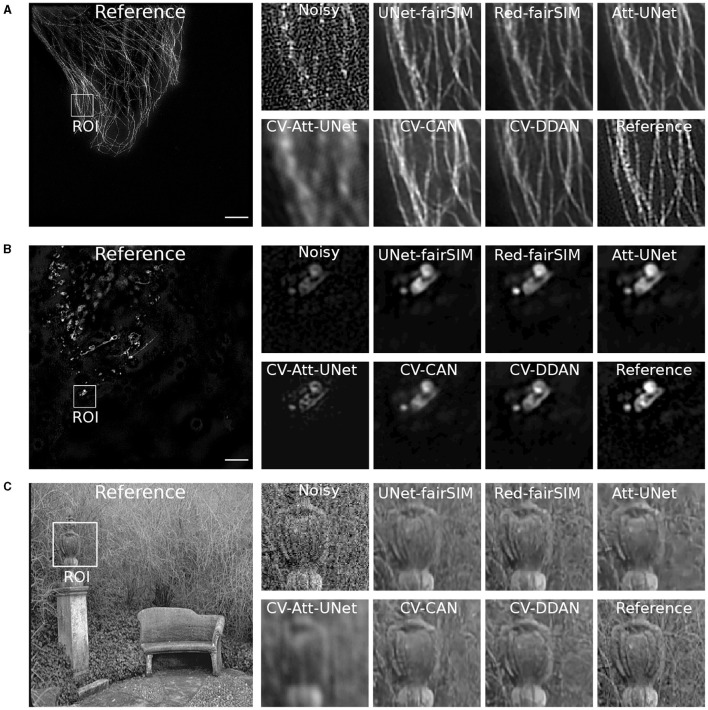
Three test samples from datasets Tubulin **(A)** and Vesicles **(B)** and BSD **(C)** are shown in this figure. Both **(A, B)** contain the reference SR-SIM image along with the resultant denoised ROIs which are extracted from the respective full-size denoised images of all the models. The extracted ROIs are upsampled to 300 × 300 from 100 × 100 pixels. SR-SIM images scale bar: 4μ*m*.

**Figure 8:**
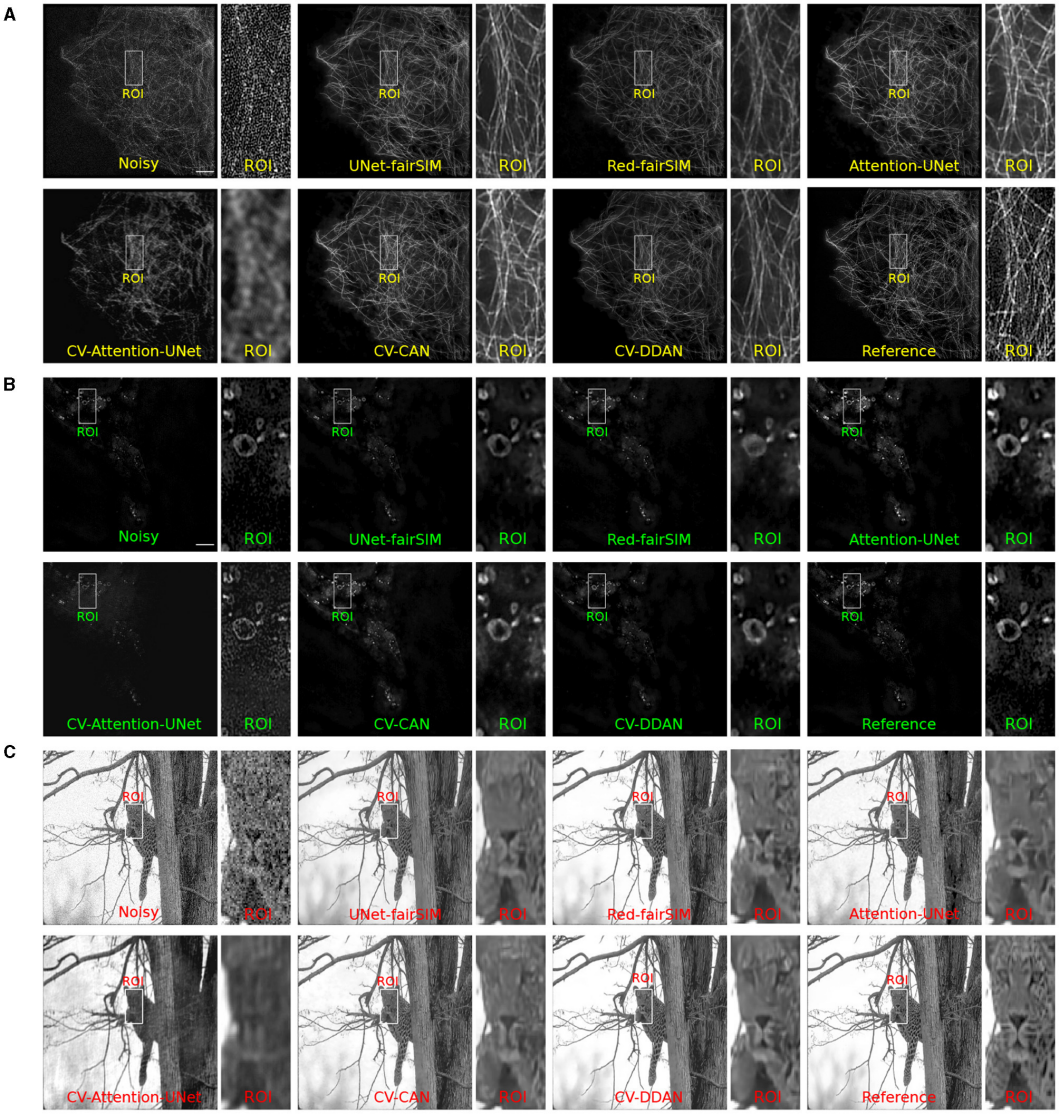
Results of test samples of Tubulin **(A)**, Vesicle **(B)**, and BSD **(C)** datasets are shown in this figure. The first two blocks show the results of Tubulin and Vesicle SR-SIM images. The first row in each block contains the noisy along with denoised images of RV-CNN based models whereas, the second row in each block shows the images denoised by CV-CNN based model together with the reference images. The odd columns present the full-size outputs of all the methods and the even number columns display the region of interest (ROI) extracted from the respective image. The cropped ROIs of size 50 × 100 pixels are upsampled to 100 × 200 for visualization. SR-SIM images scale bar: 4μ*m*.

To evaluate the real-valued and complex-valued networks for the image denoising task, we first trained several state-of-the-art real-valued denoising networks, such as UNet-fairSIM, Red-fairSIM (Shah et al., 2021), and Attention-UNet (Oktay et al., 2018), on the aforementioned datasets for three trials each. The quantitative and visual results of these real-valued networks are shown in Table 1 and Figures 6–8. To summarize the results of the real-valued networks, it is worth mentioning that the Red-fairSIM provides visually and quantitatively (i.e., individually and collectively) superior results for all three datasets among the real-valued denoising networks employed in this study.

**Table 1:** Mean PSNR and SSIM values of three runs along with standard deviations (STD) of all methods calculated on complete test data of Tubulin, Vesicle, and BSD datasets.

	**Mean PSNR (STD) and SSIM (STD) values of different methods**					
	**Tubulin dataset**		**Vesicle dataset**		**BSD dataset**	
	**1st run 2nd run 3rd run PSNR** ±**STD**	**1st run 2nd run 3rd run SSIM** ±**STD**	**1st run 2nd run 3rd run PSNR** ±**STD**	**1st run 2nd run 3rd run SSIM** ±**STD**	**1st run 2nd run 3rd run PSNR** ±**STD**	**1st run 2nd run 3rd run SSIM** ±**STD**
fairSIM	23.61 23.61 23.61 23.61 ± 0.00	0.29 0.29 0.29 0.29 ± 0.00	35.10 35.10 35.10 35.10 ± 0.00	0.86 0.86 0.86 0.86 ± 0.00	18.99 18.99 18.99 18.99 ± 0.00	0.28 0.28 0.28 0.28 ± 0.00
Red-fairSIM	27.97 27.98 27.89 27.94 ± 0.04	0.71 0.70 0.70 0.70 ± 0.00	38.43 39.01 38.95 **38.79** **±0.26**	0.89 0.88 0.89 0.88 ± 0.00	29.71 29.98 29.74 **29.81** **±0.12**	0.83 0.83 0.82 0.82 ± 0.00
UNet-fairSIM	26.80 27.82 27.92 27.51 ± 0.35	0.69 0.70 0.70 0.69 ± 0.00	37.45 37.99 37.96 37.80 ± 0.24	0.88 0.88 0.88 0.88 ± 0.00	29.56 26.39 29.06 28.33 ± 1.39	0.82 0.82 0.82 0.82 ± 0.00
Attention-UNet	27.37 28.21 27.14 27.57 ± 0.45	0.69 0.69 0.69 0.69 ± 0.00	37.50 37.64 37.58 37.57 ± 0.05	0.88 0.88 0.88 0.88 ± 0.00	27.25 27.67 28.93 27.95 ± 0.71	0.82 0.80 0.82 0.81 ± 0.00
CV-Attention-UNet	26.81 27.29 26.99 27.02 ± 0.19	0.62 0.63 0.62 0.62 ± 0.00	35.70 36.12 35.12 35.70 ± 0.40	0.83 0.83 0.82 0.82 ± 0.00	21.21 26.52 22.70 23.47 ± 2.23	0.63 0.64 0.64 0.63 ± 0.00
CV-CAN	27.44 27.86 27.50 27.60 ± 0.18	0.71 0.71 0.70 0.70 ± 0.00	36.56 36.79 37.23 36.86 ± 0.27	0.88 0.88 0.88 0.88 ± 0.00	28.59 28.87 28.68 28.71 ± 0.11	0.82 0.82 0.81 0.81 ± 0.00
CV-DDAN	28.13 28.02 27.92 **28.02** **±0.08**	0.73 0.73 0.73 **0.73** **±0.00**	37.83 37.48 37.59 37.63 ± 0.14	0.89 0.89 0.89 **0.89** **±0.00**	29.21 29.27 29.68 29.38 ± 0.20	0.83 0.83 0.83 **0.83** **±0.00**

The highest mean PSNR and SSIM values of the three attempts are indicated in bold.

To investigate the potential of CV-CNNs, we first trained a simple pure CV-CNN based method called CV-Att-UNet (i.e., explained in Section 3.3), which mimics the traditional Attention-UNet architecture for the denoising tasks. First, dummy zero imaginary values are introduced to the scalar pixel values of the input/output images to convert them from real to complex pixel values. In this setting, the CV-Att-UNet network provides almost identical results to RED-fairSIM. However, a significant drop in CV-Att-UNet performance was observed when the input and output images were transformed using the FFT approach, as illustrated in Table 1. Similarly, denoised images of the CV-Att-UNet reveal that the noise has been reduced, but the structural information of the object is also missing from the resulting images, as shown in Figures 6–8. This investigation indicates that the pure CV-CNN-based architecture, such as CV-Att-UNet, fails to completely denoise and restore the images in the frequency domain, particularly with high noise levels.

To utilize the CV-CNNs more effectively, we designed two novel CV-CNN architectures, CV-CAN (explained in Section 3.4) and CV-DDAN (explained in Section 3.5), for the image denoising task. The complex-valued layers in the CV-CAN are used in a serial scheme to create a compact network. The quantitative findings of the CV-CAN model are equivalent to the real-valued networks for the Tubulin dataset shown in Table 1. However, we noticed a drop in the PSNR and SSIM values (i.e., see Table 1) for the BSD and Vesicle datasets. The CV-CAN provides superior denoised images with improved visual appearance compared to the real counterparts, as shown in the ROIs of Figures 6–8, specifically for the Tubulin and Vesicle datasets.

**Figure 9:**
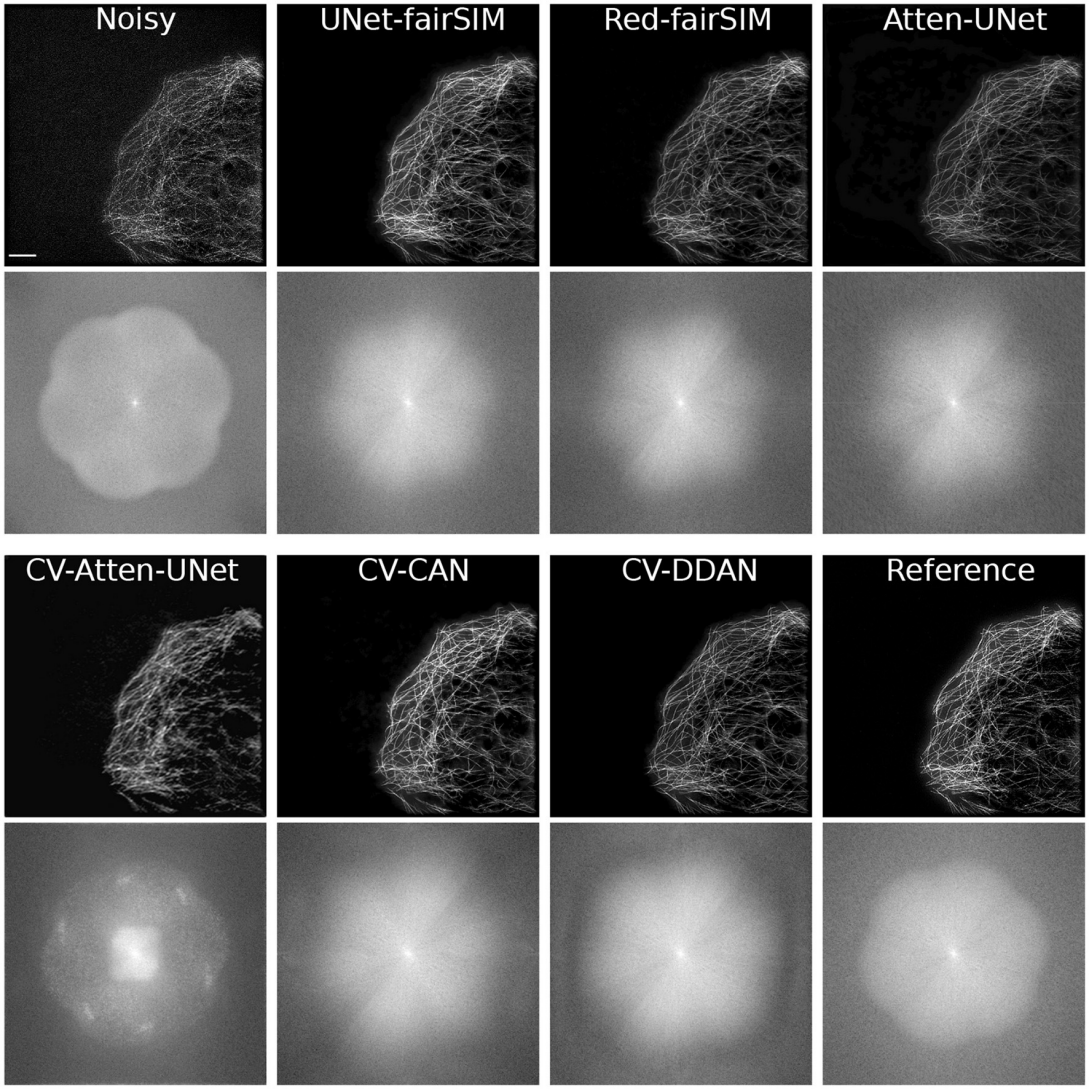
This figure shows the results of test samples of the Tubulin dataset along with the FFT spectrum. The first and third rows show the results of the spatial output of the Tubulin images. The second and fourth rows show the Fourier spectrum of the corresponding images. SR-SIM images scale bar: 4μ*m*.

The architecture of CV-DDAN is also a combination of real and complex-valued based CNN layers. The architecture of CV-DDAN is composed of real and complex-valued branches connected in a parallel scheme with a summation-based operator in the final block. This network requires two concurrent inputs: the spatial domain input for the real branch and the frequency domain input for the complex branch. The denoised ROIs of CV-DDAN in Figures 6–8 show more promising results than the other networks for both microscopic SR-SIM and conventional BSD datasets. The quantitative findings on the Tubulin dataset clearly demonstrate that the proposed CV-DDAN outperforms all other image denoising methods in terms of the individual and collective average PSNR and SSIM values as shown in Table 1. However, in the case of the Vesicle and BSD datasets, a modest decline in average PSNR values can be noticed in the Table 1, though the CV-DDAN is still superior in terms of average SSIM values. Similarly, the Fourier spectrum of all denoised images shows that the FFT spectrum of both CV-CAN and CV-DDAN networks is dense and visually similar to the FFT spectrum of the reference image (see Figure 9). This implies that our proposed complex-valued-based approaches preserve more high-frequency information than their real-valued counterparts.

### 4.2 Super-resolution

The results of the CV-SRN architecture (explained in Section 3.6) are compared with a real-valued super-resolution UNet (SR-UNet). The SR-UNet is a modified form of the conventional UNet (Ronneberger et al., 2015) in that it is extended by an upsampling block. The upsampling block consists of upsampling and convolution layers. The SR-UNet works with the spatial domain inputs and outputs. In contrast, the CV-SRN takes a frequency domain input and produces a twofold spatial high-quality super-resolved output. In the case of SR-SIM reconstruction, 15 raw SIM images of size 512 × 512 (width × height) pixels are combined to compute the average projection image. The input image is transformed into a complex domain by the FFT transformation.

**Figure 10:**
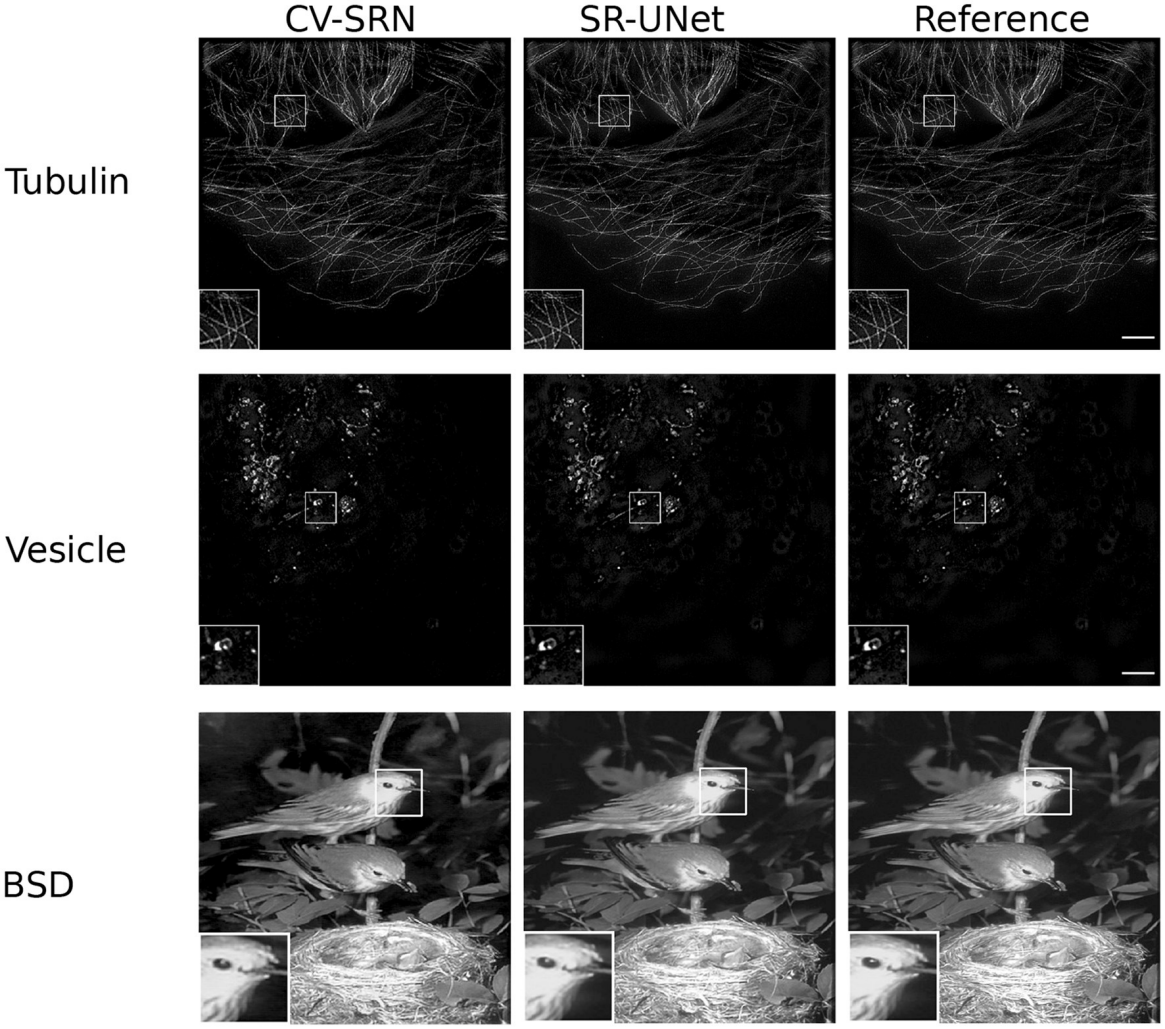
Results of test samples from the Tubulin (first row), Vesicle (second row), and BSD (third row) datasets are shown in this figure. The first, second, and third columns present the results of the CV-SRN and SR-UNet methods. The third column shows the reference images. The cropped and zoomed regions of interest (ROIs) of size 100 × 100 pixels are upsampled to 200 × 200 pixels and shown on the lower left corner of the full-size image. SR-SIM images scale bar: 4μ*m*.

The experimental results in Figure 10 and Table 2 show that the CV-SRN can successfully carry out the super-resolution task, i.e., that it can generate a double spatial dimension high-resolution output image from the high SNR low-dimensional image. The visual findings demonstrate good quality structures almost identical to the real-valued network, however, the mean PSNR and SSIM values in Table 2 are smaller than for the real-valued counterpart. However, the CV-SRN is limited to super-resolution tasks and fails to yield presentable results in the case of joint denoising and super-resolution tasks from low-SNR SIM images. This indicates again the limitations of pure CV-CNN networks (similar to the CV-Att-UNet network in Section 4.1) for general image restoration tasks.

**Table 2:** Mean PSNR and SSIM values along with standard deviations (STD) calculated over the test samples of the BSD, Tubulin, and Vesicle datatests for super-resolution tasks.

	**Mean PSNR (STD) and SSIM (STD) values of different methods**			
	**CV-SRN**		**SR-UNet**	
	**PSNR (STD)**	**SSIM (STD)**	**PSNR (STD)**	**SSIM (STD)**
Tubulin dataset	46.37 (0.60)	0.95 (0.003)	40.73 (2.10)	0.97 (0.00)
Vesicle dataset	60.05 (1.15)	0.98 (0.00)	61.75 (1.08)	0.99 (0.00)
BSD	34.54 (1.90)	0.94 (0.00)	36.37 (1.95)	0.98 (0.00)

### 4.3 Knowledge transfer

**Figure 11:**
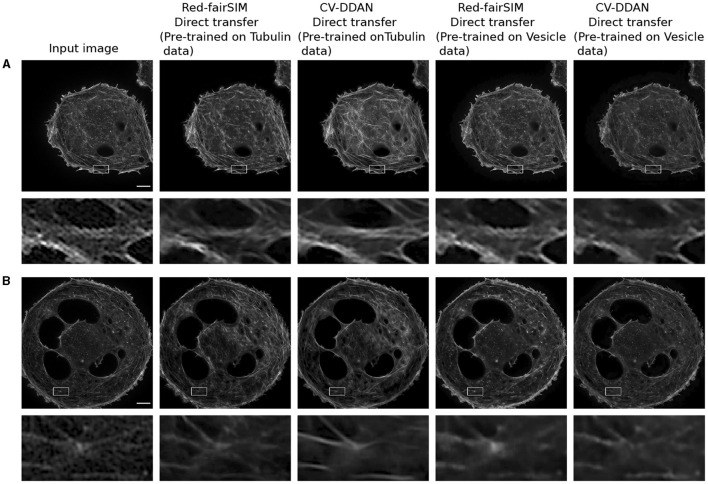
This figure depicts the result of LSECs with Phalloidin (actin) staining (i.e., **A, B**) images denoised by direct transfer. The first column illustrates the input images. The second and fourth columns present the denoised outcomes of Phalloidin actin produced by the Red-fairSIM models pre-trained on the Tubulin and Vesicle datasets. The third and fifth columns portray the denoised images of the CV-DDAN methods trained on Tubulin and Vesicle datasets. The second and fourth rows show the zoomed ROIs extracted from the actual denoised image for demonstration purposes. Scale bar: 4μ*m*.

**Figure 12:**
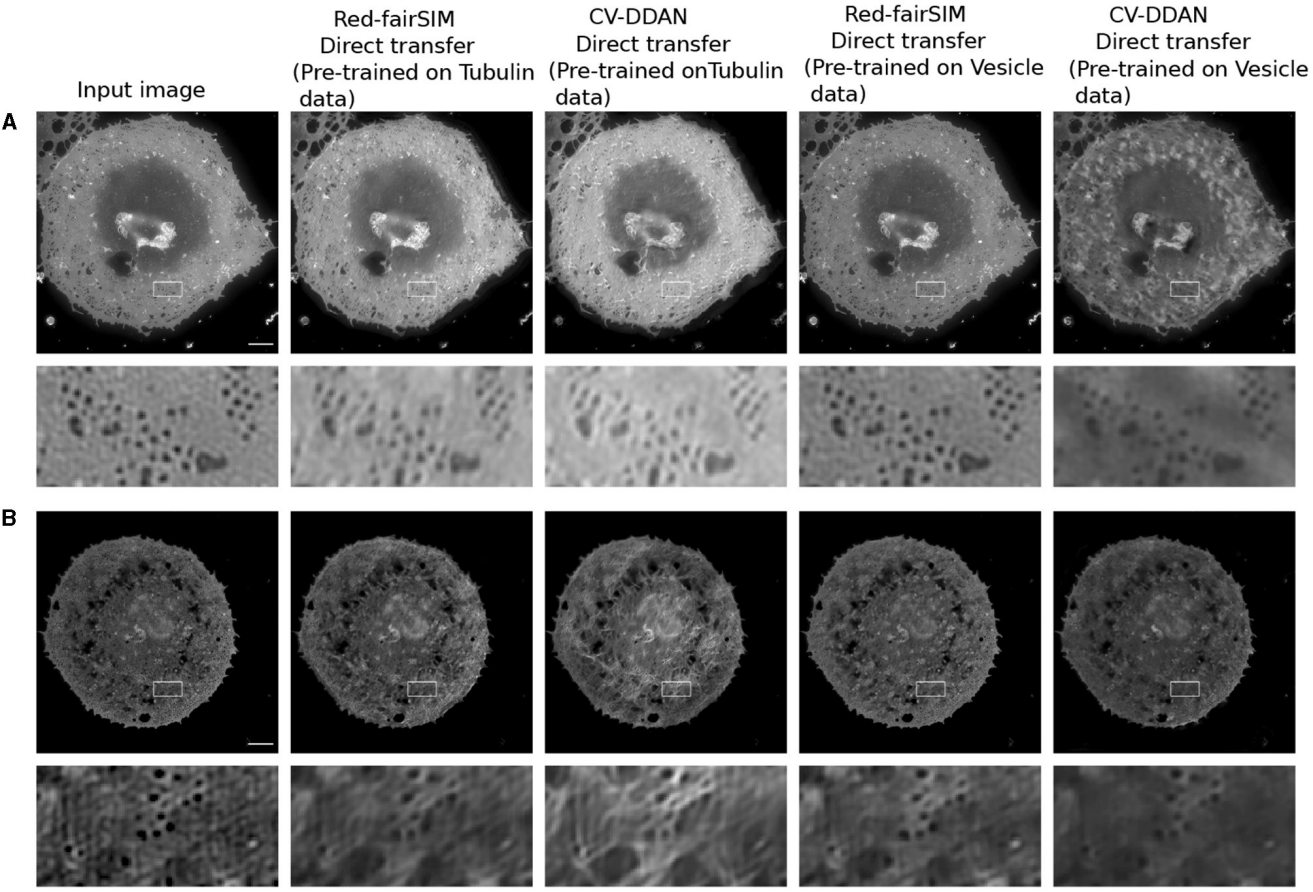
Result of the direct transfer strategy on the LSECs stained with BioTracker membrane (i.e., **A, B**). The first column shows the input images. The second and fourth columns display the outcomes of the Red-fairSIM networks pre-trained on the Tubulin and Vesicle datasets. The third and fifth columns depict the denoised images by the CV-DDAN method. The second, and fourth rows show the zoomed ROIs extracted from the corresponding above given images. Scale bar: 4μ*m*.

CV-CNNs are known for their strong ability to generalize well (Lee et al., 2022). In this work, we therefore also explore the generalization power of RV-CNNs and CV-CNNs on previously unseen biological structures in a direct-transfer setting. Direct transfer refers to the ability of pre-trained models to generalize on unseen new test data from a closely related domain without any retraining or fine-tuning. For our direct-transfer experiment with selected RV-CNN and CV-CNN architectures (Red-fairSIM and CV-DDAN), liver sinusoidal endothelial cells (LSECs) stained with phalloidin (actin) and BioTracker membrane structures were collected via total internal reflection fluorescence – structured illumination microscopy (TIRF-SIM) (Barbieri et al., 2021) as additional test data. The results of these tests are shown in Figures 11, 12.

A closer look at Figures 11A, B shows that the previously trained CV-DDAN model on the Tubulin dataset provides visually more convincing denoised images of LSECs stained with phalloidin (actin) probes than the Red-fairSIM model. The CV-DDAN trained on the Vesicle dataset cannot fully recover the structure compared to the Red-fairSIM model, as shown in Figure 11, but still produces adequate results by suppressing the noise, as shown in Figure 11 (see full fifth column). Similarly, these pre-trained models are able to produce denoised images of the LSECs BioTracker membrane samples as shown in Figure 12. The Red-fairSIM model pre-trained on Vesicle data shows very compelling results among all other pre-trained models as illustrated in Figures 12A, B. However, the images of LSEC stained with BioTracker membrane samples denoised by the CV-DDAN pre-trained with the Vesicle dataset appear suppressed compared to other methods. Overall, we can clearly see from the results of the direct transfer approach that the pre-trained models are able to reconstruct and denoise the images of the new unseen samples to some extent, despite the characteristics of the networks. The denoised images obtained by the CV-DDAN seem to be more promising and superior in some images, but not completely.

## 5 Discussion

During this work, we thoroughly investigated CV-CNNs for denoising and super-resolution problems. The findings of this study provide sufficient use cases of CV-CNNs for various datasets with different noise levels and noise types. CV-CNNs were not completely investigated previously for image restoration tasks, particularly image denoising in the frequency domain. Here, we demonstrated the effectiveness of CV-CNNs for multiple image restoration tasks. We also suggested multiple approaches for efficiently deploying CV-CNNs for the image denoising and super-resolution. In addition, we addressed the following questions: 1. Is it possible to denoise and super-resolve the images entirely in the frequency domain or the complex domain (i.e., after the FFT transformation) using CV-CNNs? 2. Do CV-CNNs outperform RV-CNNs in terms of generalization performance when applied to unseen SR-SIM modalities? To answer these questions, we trained multiple RV-CNN and CV-CNN based architectures on three datasets. We demonstrated that the pure CV-CNN based architecture (CV-Atten-UNet) cannot outperform its real counterparts on its own. However, the fusion of spatial and frequency information in our parallel scheme (CV-DDAN) provides better results than the other approaches to a certain extent. The visual results clearly show that the CV-DDAN approach outperforms its real and complex-valued image denoising counterparts on all tested datasets. While the quantitative results are better for the tubulin dataset regarding both PSNR and SSIM, only the mean SSIM values improve for the vesicle and BSD datasets. In contrast, the CV-CAN (serial scheme) produces only comparable results to real-valued SOTA denoising networks such as Red-fairSIM, UNet-fairSIM, and Attention-UNet. Overall, the fusion of frequency and spatial learning in CV-CNN based models is crucial to enhance the recovery of signals in both domains, particularly to improve the performance in the high-frequency region, as shown in Figure 9.

Similarly, the results of CV-SRN show that the CV-CNNs perform well for the super-resolution task for all three datasets, as shown in Figure 10. The visual results clearly indicate that the network designed entirely with CV-CNNs alone can generate good-quality super-resolved images from the noiseless inputs, but it cannot reconstruct high-quality super-resolved output images from the images acquired under low SNR conditions. Furthermore, when comparing the generalization ability based on the direct transfer strategy, the RV-CNN and CV-CNN methods yield mostly comparable results. The effectiveness of model generalization is influenced by the diversity of the datasets and the complexity of the architectures. Therefore, it is very likely that one method will outperform the other on certain examples while struggling on others, which was indeed the case in our experiments.

A recent study (Quan et al., 2021) used CV-CNNs and proposed a CDNetwork (Complex-valued Denoising Network) to denoise the images in the complex domain by adding constant imaginary values to the pixel values in the spatial domain instead of transforming the images by FFT algorithm. Similarly, the authors in Rawat et al. (2021) introduced CVMIDNet, a CV-CNN based method to eliminate Gaussian noise from chest X-ray images. In Pham et al. (2021), authors presented the Complex Fourier Network to generate complex filters for the denoising of small size images of SET12 and CBSD68 datasets. Dedmari et al. (2018) reconstructed Magnetic Resonance Imaging (MRI) by training a complex dense fully convolutional neural network (CDFNet). These above-mentioned proposed CV-CNN based architectures were either trained on specific data (i.e., MRI or X-ray, etc) or on very small-size images. Shao et al. (2023) designed an uncertainty-guided hierarchical frequency domain Transformer to learn both low and high-frequency components using a combination of real-valued CNNs and vision Transformer rather than CV-CNNs.

The authors in Qiao et al. (2021) exploit the learning of frequency features of SR-SIM images in the spatial domain of the channel attention module. Their learning of spatial and frequency information was purely based on RV-CNNs. Recently, Liu et al. (2023) proposed a dual-domain learning strategy for end-to-end SIM reconstruction using CV-CNNs. Their novel architecture involves the repetitive conversion of feature maps from the frequency domain to the spatial domain prior to the addition operation with spatial features in each dual-domain residual block. Due to this repeated transformation of features, this block-wise approach could lead to significantly higher computational cost and also fail to exploit the full potential of CV-CNN layers (Liu et al., 2023). In contrast, in our architectures, the spatial and frequency branches are designed in a completely parallel scheme with all of their corresponding components to cover a wide range of local and global spatial and frequency features. Moreover, our approach is not limited to the end-to-end reconstruction of SR-SIM, but provides a versatile method for denoising images of all kinds.

The incorporation of complex operations in the CV-CAN, CV-DDAN, and CV-SRN results in the addition of phase information, which led to easier optimization (Nitta, 2002), better performance, and improved generalization ability. The CV-CNNs are mostly dense and contain more training parameters than the RV-CNNs due to their characteristics. According to the evaluated training times, the CV-DDAN requires twice the time of its real-valued counterpart, such as Red-fairSIM. However, excellent performance is more important than computational efficiency in the field of biomedical sciences.

## 6 Conclusions

We demonstrated that our proposed CV-CNN based serial (CV-CAN) and parallel (CV-DDAN) architectures provide mostly denoising results which are superior to purely real-valued approaches. Similarly, the CV-SRN produces acceptable results for the super-resolution task. We have also remarked that the CV-SRN is limited to super-resolution tasks and fails when used in conjunction with denoising. The CV-Atten-UNet is able to suppress and eliminate noise from high frequency components, but an overall image degradation is observed throughout the denoising process. This raises concerns about the ability of pure CV-CNNs to perform image denoising. Therefore, a main result of our findings is that the fusion of spatial and frequency information by simultaneously processing spatial and complex features in architecture such as CV-DDAN is the only way forward to improve the image quality and reduce the loss of high frequency information in the Fourier spectrum. In addition, the real and complex-valued attention gates were also useful for effectively learning local and global frequency features.

## Data Availability

Publicly available datasets were analyzed in this study. This data can be found here: http://gigadb.org/dataset/102461.

## Footnotes

^1^Although the CV-CAN and CV-DDAN networks converge faster, typically after 40 epochs than real-valued CNNs, which take 75 epochs, all models were trained and monitored for 100 epochs to ensure a fair comparison. From these 100 epochs, the best performing models are selected. During the training process, the optimal learning rate was set to 1 × 10^−4^ to train all networks.
